# Reduction in thermal stress of marine copepods after physiological acclimation

**DOI:** 10.1093/plankt/fbac017

**Published:** 2022-04-08

**Authors:** Enric Saiz, Kaiene Griffell, Manuel Olivares, Montserrat Solé, Iason Theodorou, Albert Calbet

**Affiliations:** INSTITUT DE CIÈNCIES DEL MAR (ICM–CSIC), PG. MARíTIM DE LA BARCELONETA 37-49, BARCELONA, CATALONIA 08003, SPAIN; INSTITUT DE CIÈNCIES DEL MAR (ICM–CSIC), PG. MARíTIM DE LA BARCELONETA 37-49, BARCELONA, CATALONIA 08003, SPAIN; INSTITUT DE CIÈNCIES DEL MAR (ICM–CSIC), PG. MARíTIM DE LA BARCELONETA 37-49, BARCELONA, CATALONIA 08003, SPAIN; INSTITUT DE CIÈNCIES DEL MAR (ICM–CSIC), PG. MARíTIM DE LA BARCELONETA 37-49, BARCELONA, CATALONIA 08003, SPAIN; INSTITUT DE CIÈNCIES DEL MAR (ICM–CSIC), PG. MARíTIM DE LA BARCELONETA 37-49, BARCELONA, CATALONIA 08003, SPAIN; INSTITUT DE CIÈNCIES DEL MAR (ICM–CSIC), PG. MARíTIM DE LA BARCELONETA 37-49, BARCELONA, CATALONIA 08003, SPAIN

**Keywords:** temperature, thermal performance curves, Q10, oxidative stress, *Paracartia grani*

## Abstract

We studied the phenotypic response to temperature of the marine copepod *Paracartia grani* at the organismal and cellular levels. First, the acute (2 days) survival, feeding and reproductive performances at 6–35°C were determined. Survival was very high up to ca. 30°C and then dropped, whereas feeding and fecundity peaked at 23–27°C. An acclimation response developed after longer exposures (7 days), resulting in a decline of the biological rate processes. As a consequence, Q_10_ coefficients dropped from 2.6 to 1.6, and from 2.7 to 1.7 for ingestion and egg production, respectively. Due to the similarity in feeding and egg production thermal responses, gross-growth efficiencies did not vary with temperature. Respiration rates were less sensitive (lower Q_10_) and showed an opposite pattern, probably influenced by starvation during the incubations. The acclimation response observed in the organismal rate processes was accompanied by changes in body stoichiometry and in the antioxidant defense and cell-repair mechanisms. Predictions of direct effects of temperature on copepod performance should consider the reduction of Q_10_ coefficients due to the acclimation response. Copepod population dynamic models often use high Q_10_ values and may overestimate thermal effects.

## INTRODUCTION

The vulnerability of a species to rising temperatures associated with climate change will depend on its capacity to withstand the new conditions or to modify its phenology or geographical ranges to more suitable environments (Beaugrand, 2014; Buckley and Kingsolver, 2012). Those changes in temperature do not only include long-term gradual rises in ocean temperature, but also unusual warming events, involving rises around 1–5°C and durations of at least five consecutive days and much longer, known as marine heatwaves (Hobday *et al.*, 2016; Oliver *et al.*, 2018). Predictions of ectotherms’ response to ocean temperature changes can be derived from concepts like thermal niche breadths and critical thermal maxima, often estimated through survival experiments. Before reaching the temperatures at which survival is challenged, however, the actual competence of the species to persist and support population growth under new environmental conditions might be better reflected by the response of relevant physiological traits, which may exhibit differences in thermal susceptibility and structure hierarchically in their overall response (Magozzi and Calosi, 2015; Pallarés *et al.*, 2020; Pan *et al.*, 2021). Not only physiological sensitivity is relevant, but also other factors like exposure time and acclimation capacity need to be considered for assessing species’ vulnerability to changing temperature conditions (Williams *et al.*, 2008; Schulte *et al.*, 2011).

In the case of planktonic copepods, conceivably the most abundant metazoans in the ocean and a key group in the functioning of marine pelagic systems, the study of temperature as a key factor affecting species physiological performance, body size and distribution has a long tradition (e.g. Anraku, 1964; Mclaren, 1965). The use of thermal performance curves to assess copepod vulnerability has been applied to understand species distribution (Halsband-Lenk *et al.*, 2002; Holste and Peck, 2006) and has recently received renewed interest in relation to climate-induced changes in the environment (Peck *et al.*, 2015; Sasaki *et al.*, 2019; Sasaki and Dam, 2019). Besides the organismal survival response, most of these studies have commonly focused on only one or two functional traits and have not covered a broader scope. Thus, Halsband-Lenk *et al.* (2002) reported that the latitudinal distribution of congeneric copepod species might be related to differences in the thermal tolerance of survival, post-embryonic development and fecundity. More recently, Alcaraz *et al.* (2014) proposed that the mismatch between the thermal responses of feeding and respiration in the calanoid *Calanus glacialis* could explain the upper limit of the thermal niche for this species in the Arctic. Further evidence by Grote *et al.* (2015) seemed to confirm this metabolic mismatch for *C. glacialis* with increasing temperatures. Unfortunately, this type of research contemplating the interaction of multiple thermal trait responses is not commonly addressed in marine copepods.

In this study, our goal was to obtain a more comprehensive view of the direct effects of temperature on copepod performance by considering this multiple response approach at different organization levels. We focus on intragenerational effects, particularly the response of adult females to temperature changes at the scale of several days. We hypothesized that different copepod physiological traits may have different thermal sensitivities, and therefore their interaction may govern the overall performance. We have also addressed the effect of exposure time on thermal stress impact and the development of the acclimation response, defined as intra-generational active phenotypic plasticity (Havird *et al.*, 2020). We predict that prolonged exposure time will influence the copepod’s phenotypic response, as a result of acclimation effects, involving a decline in metabolic activation energies and Q_10_ coefficients that may partially or completely compensate the acute, passive thermal effects. At the organismal level, temperature-driven changes in the vital rates may also alter the homeostatic balance resulting in altered body stoichiometry. To do that, first we determined the thermal performance curves of the marine calanoid copepod *Paracartia grani* over a broad temperature range (6–35°C) and defined the thermal limits for survival, feeding and reproduction. Then, we determined the Q_10_ coefficients and studied the acclimation response of the copepod’s feeding, reproductive and metabolic activities after short- (2 days) and medium-term (7 days) exposures, together with the possible subsequent changes in body stoichiometry (Mathews *et al.*, 2018). In addition, since thermal stress likely increases the production of reactive oxygen species, which may destabilize the cellular homeostasis, we also expect copepods to show biochemical modifications at the cell level to counteract them and reduce oxidative stress. Therefore, we quantified the lipid peroxidation (LPO) levels in the copepods as a measure of oxidative cell damage, as well as several biomarkers of the copepod enzymatic antioxidant defense and cell repair capacity (Glippa *et al.*, 2018). Particularly, we determined the enzyme activity levels of (i) catalase (CAT), a prominent reactive oxygen species scavenger that converts hydrogen peroxide to water (Monaghan *et al.*, 2009), (ii) glutathione S-transferase (GST), an enzyme involved in detoxification processes by catalytic conjugation of glutathione, one of the most active antioxidant compounds that neutralize reactive oxygen species (Monaghan *et al.*, 2009) and (iii) carboxylesterase (CbE), a biomarker that integrates a group of hydrolytic enzymes involved in the metabolism of xenobiotic as well as endogenous compounds (Nos *et al.*, 2021). Finally, it is well known that rising temperatures result, at least within thermal tolerance limits and short-time scales, in the enhancement of copepod swimming activity (Svetlichny *et al.*, 2017). However, it is uncertain how longer exposure times to thermal stress may affect the copepod neuromuscular transmission and locomotor capacity. A decrease in locomotor capacity may result in less efficient feeding or higher predation risk. For this reason, in our biochemical analyses we also determined the activity of acetylcholinesterase (AChE), an enzyme involved in the regulation of the neural impulse (Pallarés *et al.*, 2020).

## METHOD

### Parental population of *P. grani*

The specimens of the copepod *P. grani* originated from our culture, kept in the laboratory for >10 years at 19°C on a diet of the flagellate *Rhodomonas salina* (Saiz *et al.*, 2015). The copepod cohorts needed for the experiments were obtained from fresh eggs (<24 h) siphoned out from the bottom of the rearing tanks and transferred to 20-L polycarbonate containers filled with 0.1-μm filtered seawater for hatching. The cohorts were reared in a temperature-controlled room (19°C) with a 10:14 light:dark cycle and in excess of *R. salina*.

### Thermal performance curve experiments

Two experiments were carried out to assess the copepod thermal performance curves at different time scales. In the first experiment, we determined the survival of adult females (<10 days since molting) after short-term exposure (2 days) to temperatures between 6 and 35°C. To do that, five baths (ca. 40 L capacity) provided with circulating pumps, TECO temperature conditioners and HOBO temperature data loggers were set-up. For the most extreme temperatures, either an aquarium heater or a lab cooling unit was used. We conducted three independent assays covering the following groups of temperatures: (a) 10.9, 15.4, 19.4, 25.6 and 30.4°C, (b) 5.7, 17.6, 19.5, 22.6 and 34.7°C and (c) 19.7, 27.4 and 32.5°C; we included the parental temperature (nominal 19°C) in all the assays.

The incubations were carried out under two food treatments: starvation (filtered seawater) and excess of food. The food treatment consisted of a suspension of ca. 650 μg C L^−1^ (ca. 2500 cells mL^−1^) of the heterotrophic dinoflagellate *Oxyrrhis marina*, which is well above satiation levels (>350 cells mL^−1^, Calbet *et al.*, 2007). *O. marina* was chosen as prey since it is motile and would not require periodically stirring to avoid settling. For each treatment, groups of 10 females were pipetted into triplicated 610-mL (filled up to the neck; 590 mL capacity) screw-cap bottles, which were then capped and placed in the corresponding baths. After 24 h, the copepods were collected onto a submerged sieve, checked for survival and then returned to the bottles, which had been refilled either with filtered seawater or fresh *O. marina* suspension. After an additional incubation of 24 h, the bottles were again sampled and copepod survival noted. The entire contents of the bottles with food were subsequently filtered onto a 20-μm sieve to collect eggs and fecal pellets to assess their production rates during this second incubation. Eggs and pellet samples were preserved in 2% Lugol’s solution for posterior counting.

In the second experiment, the short- (2 days) and medium-term (7 days) thermal responses of *P. grani* were compared. The general procedure was similar to that mentioned before, although we focused on a narrower thermal range within the tolerance limits previously determined (10.1, 16.0, 19.1, 22.0, 25.1 and 28.0°C). All incubations in the second experiment were conducted in excess of food (ca. 650 μg C L^−1^ of *O. marina*), with 12 adult females in each triplicated bottle. Food was replenished every 2 days by siphoning out most of the bottle contents through a 100-μm mesh, and then refilling it with fresh *O. marina* suspension. Egg and fecal pellet production rates were determined after 2 and 7 days of exposure following the procedure described before. For that purpose, on the previous day (i.e. the first and sixth days of incubation) the bottle contents were gently sieved through a submerged sieve, the copepods were examined and kept momentarily aside, then the bottles were rinsed and refilled with fresh food suspensions and finally, the copepods were transferred back.

### Short-term and medium-term biochemical and physiological responses of *P. grani*

We investigated the responses of biochemical indicators and physiological activities to moderate changes in temperature. We exposed the parental copepod population (19°C) to 16.1, 21.8 and 24.8°C, and assessed the effects of short- (2 days) and medium-term (7 days) thermal exposures; the nominal 19°C (18.9°C) was also included as control treatment. The copepod feeding, egg production and respiration rates were determined, as well as the changes in Q_10_ coefficients after the different acclimation periods. Moreover, we measured the changes in stoichiometric ratios (C:N, C:P and N:P) and key biomarkers of oxidative stress in the copepods exposed to the different treatments.

The general experimental design was as follows. On the start day, for each temperature we prepared triplicated 4-L Nalgene bottles filled with a suspension of ca. 2100 μg C L^−1^ of *O. marina* made with water previously conditioned to the respective temperature. We added to each 4-L Nalgene bottle a mixture of $\sim$300 females and 100 males *P. grani* from our parental (19°C) copepod stock culture, and then we left the bottles in the water baths to allow copepods experience the different thermal treatments. Every day (in one occasion 2 days) most of the 4-L Nalgene bottle contents were siphoned out through a 100-μm mesh (retaining the copepods inside) and refilled with fresh suspension of 2100 μg C L^−1^ of *O. marina* (2850 μg C L^−1^ in the 2-day refill) prepared with temperature-conditioned seawater.

Feeding and egg production incubations were started after 1 and 6 days of thermal exposure. Triplicated groups of adult females from the acclimation 4-L Nalgene bottles were transferred to 610-mL screw-cap bottles filled with *O. marina* suspensions (900 μg C L^−1^) made with temperature-conditioned water, and then left standing in the corresponding water baths. The number of individuals per bottle was inversely related to temperature (17, 15, 13 and 12 females for, respectively, the 16, 19, 22 and 25°C treatments) to prevent excessive food reduction at high temperatures, or not detectable grazing at the lowest ones. Parallel triplicated control bottles with only prey suspensions were also set to account for changes in prey concentration through the incubation. After ca. 22 h, the copepods were gently sieved, checked and counted. Then, the entire bottle contents were filtered onto a 40-μm sieve to collect the eggs, which were preserved as aforementioned, and posteriorly counted. Aliquots of the screened water were processed with a Multisizer 3 Coulter counter to determine prey volume and concentration. Ingestion rates were calculated using Frost’s equations.

Copepod respiration rates were determined from parallel incubations carried out in 0.1-μm filtered seawater. Copepods from the acclimation 4-L Nalgene bottles were taken, left to clear their guts in filtered seawater and groups of 19, 17, 15 and 13 adult females were transferred to 70-mL screw-cap bottles filled with temperature-conditioned filtered seawater. The bottle caps had a Teflon liner to avoid bubbles. Triplicated additional bottles without copepods were also incubated to serve as controls. The bottles were placed in the corresponding temperature-adjusted water baths (i.e. 16, 19, 22 and 25°C), and after ca. 22 h of incubation, the oxygen concentration was determined with an oxygen dipping probe (optodes, PreSens). Copepod respiration rates were calculated from the differences in oxygen concentration between the bottles with and without copepods. The oxygen consumption rates were converted into carbon losses using a respiratory quotient of 0.97 (Omori and Ikeda, 1984). Although the outcome of the respiration experiment suggested a clear trend, variability was noticeable (see Results) and we repeated the respiration incubations at short- and medium-term exposures with a new cohort of adults to confirm the trends observed.

Additional copepod samples were taken from the 4-L Nalgene bottles on the days of initiation of the physiological incubations (Days 1 and 6) for CNP analyses and biomarker determinations. The copepods were left to clear their guts in filtered seawater, and then narcotized with MS-222 before transferring them in groups of 15 females to pre-combusted 25-mm Whatman GF/C filters (450°C, 5 h) for C, N and P analyses. Filters for CN analysis were dried in an oven at 60°C for 48 h and then kept in a desiccator until analysis with a Flash EA1112 (Thermo Finnigan) CHNS analyzer. Filters for P analysis were frozen at −80°C and later analyzed as inorganic P after orthophosphate acid persulphate oxidation. Since the filters used for P and CN analyses were independent, the standard errors of C:P and N:P ratios were computed by error propagation (Saiz *et al.*, 2020). Moreover, for biomarker determinations, triplicated groups of 40 females were transferred with a needle into Eppendorf vials and then stored at −80°C. The determination of enzyme activities (CAT, GST, CbE using ρ-nitrophenyl butyrate as substrate and AChE) and LPO levels followed similar procedures to those described in Saiz *et al.* (2015). Kinetic protocols also included commercial purified proteins as methodology quality control, as described in Solé *et al.* (2020). The copepod samples were sonicated in 200 μL of Tris buffer (20 mM at pH 7.8), the resulting homogenate was centrifuged at 10 000 *g* for 15 min at 4°C and the supernatant (S10) was used for biochemical determinations. The total protein content in the homogenates was calculated by the Bradford method to relate activities to total protein content. Assays were carried out in duplicate aliquots at 25°C in 96-well plates using a Tecan Infinite M200 spectrofluorometer microplate reader.

Preserved females and eggs were photographed with an inverted microscope, and female prosome length and egg diameter were determined with the ImageJ software. Copepod carbon content was used to calculate weight-specific physiological rates. Carbon egg content was derived from egg size and the conversion factor provided by Saiz *et al.* (2020) for the same species (0.129 pg C μm^−3^). Carbon intake was calculated from prey volume using our factor for *O. marina* in the experiments (0.119 pg C μm^−3^), determined with the methods aforementioned. Gross-growth efficiency was calculated as the quotient between carbon-specific egg production and ingestion rates.

Physiological acclimation was assessed by comparing the Arrhenius plots and calculating the temperature coefficients Q_10_ at short- and medium-term exposures. First, simple linear regression analysis was carried out between the inverse of temperature (1/T, in Kelvin degrees) and the natural logarithm of the carbon-specific physiological rate (i.e. the Arrhenius plot) and statistical differences between slopes were determined. Then, the activation energy (E_a_) was calculated from the slopes of the linear fit as E_a_ = −slope $\times$ R, where R is the universal gas constant (8.3145 J mol^−1^ K^−1^). Finally, Q_10_ coefficients were calculated from E_a_ as:

$$ {\mathrm{Q}}_{10}=\exp \left(\frac{{\mathrm{E}}_{\mathrm{a}}}{\mathrm{R}}\times \frac{10}{{\mathrm{T}}_{\mathrm{m}}^2}\right) $$

where T_m_ is the mean for the range of temperatures over which the copepods have been exposed (i.e. between 16 and 25°C) (Raven and Geider, 1988).

### Statistics

Thermal performance curves were fitted to the model by Thomas *et al.* (2017) using the R package rTPC (Padfield *et al.*, 2021). This is a double-exponential model, which combines exponential increase and decrease functions. Other statistical analyses were conducted with Prism 7. Statistical differences in Q_10_ coefficients were assessed by comparing the slopes of the Arrhenius plots with F tests, with *P* values corrected for multiple comparisons when necessary. Otherwise noted, *P* values are two-tailed.

## RESULTS

### Thermal performance curves

**Fig. 1:**
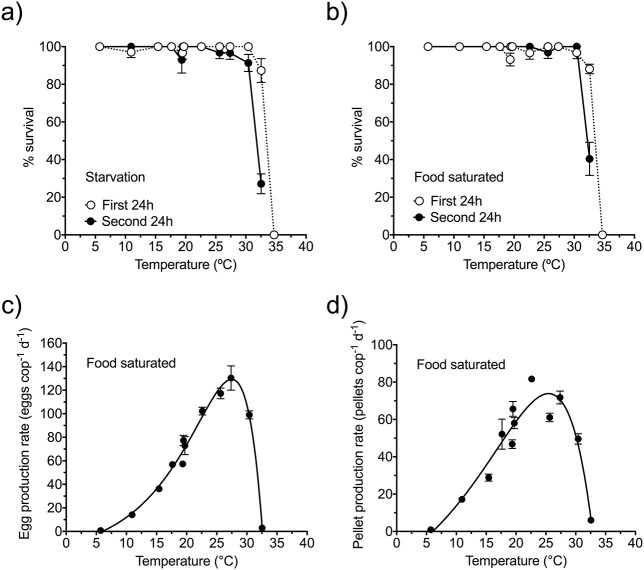
Thermal response of *P. grani* exposed for 2 days to temperatures between 5 and 35°C. (**a**) Survival in starvation, (**b**) survival in saturated food conditions, (**c**) egg production rate and (**d**) fecal pellet production rate. Egg and fecal pellet data were obtained only during the second 24-h period in the saturated food treatment. Thermal performance curves in (**c**) and (**d**) were fitted to a double-exponential model (see text). Error bars are SE.

Fig. 1a and b shows the survival curves for *P. grani* females under short-term exposure (2 days) to a broad range of temperatures (6–35°C). At 35°C all copepods died within the first 24 h of exposure regardless of food availability. Although copepod survival was still high at 33°C for the first 24 h, it drastically decreased the following day. This effect was severer when copepods were kept without food (48 h survival was 40% and 27% for starvation and food presence treatments, respectively; Fig. 1a and b). Mortality was absent or negligible below 33°C, and a (qualitative) reduction in locomotor activity was observed at 6°C. The thermal performance curves of egg and fecal production rates under short-term exposure (2 days) showed an asymmetrical dome shape. There was a steady increase from ca. 1 egg ind^−1^ day^−1^ and 1 pellet ind^−1^ day^−1^ at 6°C up to 130 eggs ind^−1^ day^−1^ at 27°C and 82 pellets ind^−1^ day^−1^ at 23°C, followed by a sharp decline to very low rates (Fig. 1c and d).

**Fig. 2:**
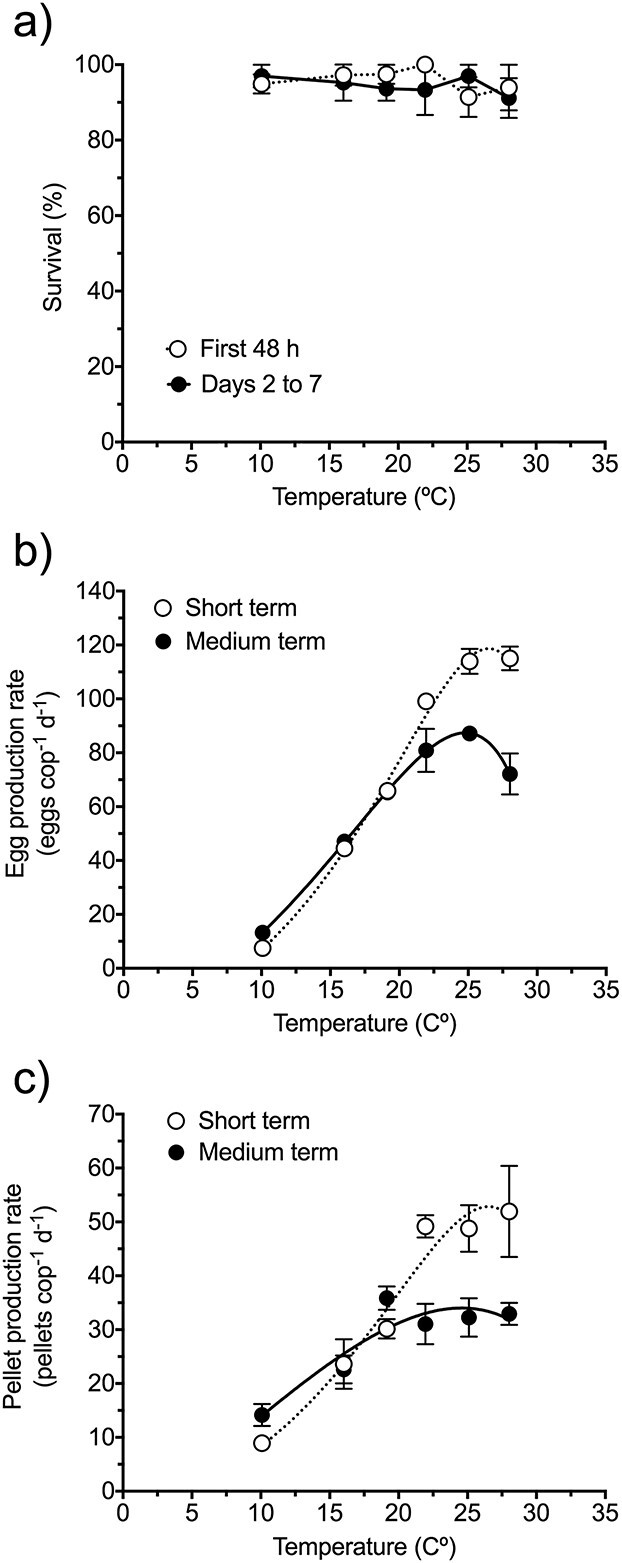
Survival (**a**), egg production rate (**b**) and fecal pellet production rate (**c**) of *P. grani* exposed for 7 days to temperatures between 10 and 28°C under saturated food conditions. “Short term” refers to the first 2 days of exposure, whereas “medium term” refers to the whole 7-day exposure period (see text for further details). Thermal performance curves in (b) and (c) were fitted to a double-exponential model (see text). Error bars are SE.

The survival of *P. grani* was not affected by the prolonged exposure (Fig. 2a) over the range of temperatures tested in the second experiment (10 to 28°C). Egg and fecal pellet production rates after short-term (2 days) exposure (Fig. 2b and c) showed a response to temperature similar to the observed in the first experiment (Fig. 1c and d) with an increase of up to ca. 25°C. However, medium-term (7 days) exposure resulted in the leveling off of both egg and fecal pellet production rates at temperatures >19°C (i.e. above the original temperature at which copepods were reared; Fig. 2b and c).

### Ingestion, egg production and respiration rates

**Fig. 3:**
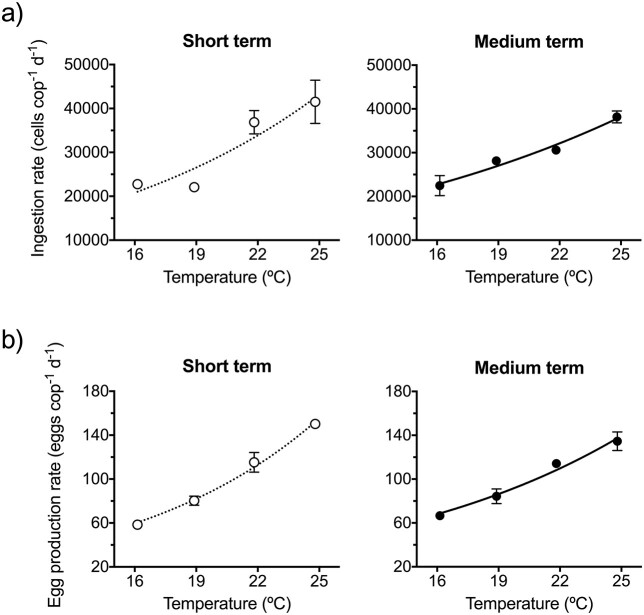
Feeding (**a**) and egg production (**b**) rates of *P. grani* after short-term and medium-term exposures at 16, 19, 22 and 25°C. Lines correspond to exponential fits. Error bars are SE.

Short-term thermal stress enhanced cell ingestion rates of *P. grani* up to values of ca. 41 500 cells ind^−1^ day^−1^ at temperatures >19°C (Fig. 3a; exponential fit, r^2^ = 0.72). Feeding rates also increased exponentially after the medium exposure (Fig. 3a; exponential fit, r^2^ = 0.87), but the range of variation of feeding rates between the lowest and the highest temperatures was 16% lower after the prolonged exposure.

Egg production rates varied between mean values of 58 and 150 eggs ind^−1^ day^−1^ after short-term exposure, and between 67 and 135 eggs ind^−1^ day^−1^ in the medium-term one (Fig. 3b). In both exposures, egg production rates increased exponentially with temperature (Fig. 3b; exponential fits, r^2^ = 0.95 and 0.90 for the short- and medium-term exposures, respectively). As with feeding rates, the range of variation between egg production rates at 16 and 25°C was lower after the prolonged exposure (26%; Fig. 3b). Moreover, there was a significant decrease in egg size with temperature, up to a 4-μm difference in diameter (i.e. 14% in volume) between 16 and 25°C (Supplementary Fig. S1; linear regression, *P* < 0.001 in both exposures; slopes did not differ, F test, *P* > 0.42).

**Fig. 4:**
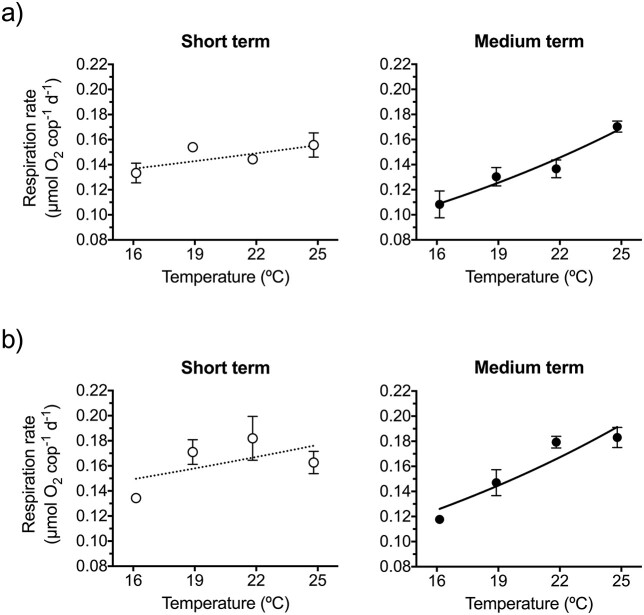
Respiration rate of *P. grani* after short- and medium-term exposures at 16, 19, 22 and 25°C. (**a**) First trial, concurrent with feeding and egg production experiments; (**b**) additional experiment with a different copepod cohort. Lines correspond to exponential fits. Error bars are SE.

In the first respiration experiment, the oxygen consumption rates of *P. grani* after short-term exposure ranged between mean values of 0.13 and 0.16 μmol O_2_ ind^−1^ day^−1^, showing poor relation with temperature (Fig. 4a; exponential fit, r^2^ = 0.29). After medium-term exposure, a more apparent trend appeared and the range of variation between the lowest and the highest temperatures increased ca. three times (Fig. 4a; exponential fit, r^2^ = 0.76). The second respiration assay resulted in a similar pattern, with clearer temperature dependence after the prolonged exposure (Fig. 4b; exponential fits, r^2^ = 0.19 and r^2^ = 0.77 after short- and medium-term exposures, respectively).

Carbon-specific ingestion and egg production rates, and the copepod respiratory carbon losses can be found in the Supplementary Information (Supplementary Figs S2 and S3), and they are also presented in the Arrhenius plots shown in the next section. Similarly, the gross-growth efficiency of *P. grani* is also shown in Supplementary Fig*.* S2, whereas its relationship with temperature will be analyzed in next section. Overall, gross-growth efficiency mean values were 0.36 ± 0.021SE and 0.39 ± 0.016SE for the short- and medium-term exposures, respectively, with no significant differences between them (*t* test, *P* > 0.29).

### Q_10_ temperature coefficients and physiological acclimation

The activation energies obtained from the Arrhenius plots and used to compute the Q_10_ coefficients are provided as supplementary in Table S1, whereas here the data will be presented only in terms of Q_10_ values.

**Fig. 5:**
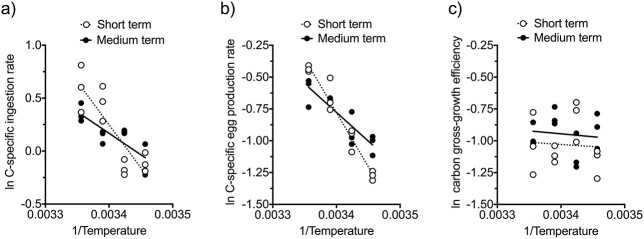
Arrhenius plots of carbon-specific ingestion rates (**a**), carbon-specific egg production rates (**b**), and carbon gross-growth efficiencies (**c**) of *P. grani* after short- and medium-term exposures at 16, 19, 22 and 25°C. Lines correspond to linear regression fits.

Medium-term exposure resulted in a statistically significant change in the slope of the Arrhenius plot for *P. grani* feeding rates compared with the short-term exposure (Fig. 5a; F test, *P* = 0.031). This change in slope (less pronounced) resulted in a decrease in the Q_10_ coefficient for feeding from 2.6 after short-term exposure to 1.6 after the prolonged acclimation (7 days). Carbon-specific egg production rates also showed a similar pattern (Fig. 5b), with a significant decline in the slope of the Arrhenius plot after the prolonged thermal exposure F test, *P* < 0.001). This variation resulted in a shift of the Q_10_ coefficients from 2.7 at short-term to 1.7 at medium-term exposure. Conversely, carbon gross-growth efficiency did not show any significant relationship with temperature neither at short- nor at medium-term exposures, with a flat response in the Arrhenius plot (Fig. 5c; linear regression analysis, *P* > 0.70 in both cases).

**Fig. 6:**
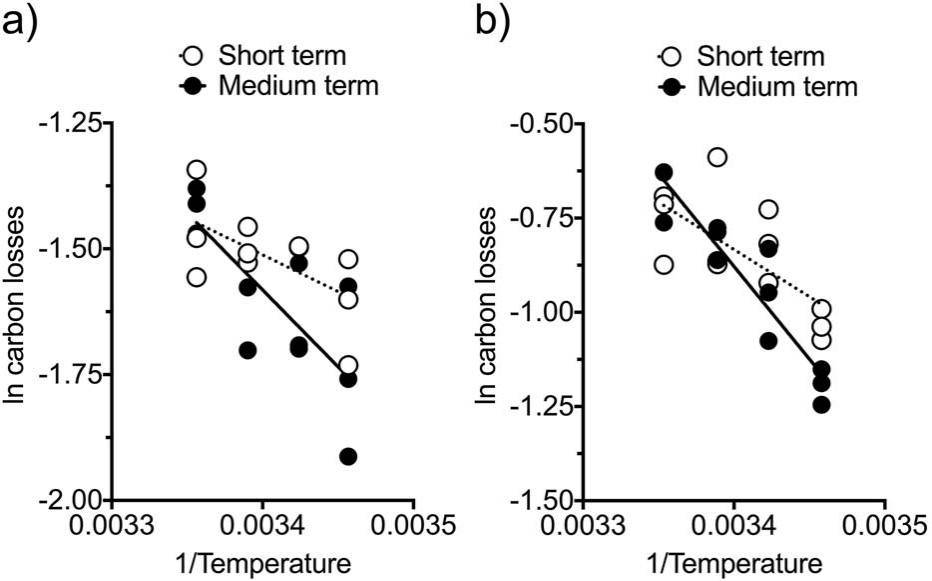
Arrhenius plots of *P. grani* respiratory carbon losses after short- and medium-term exposures at 16, 19, 22 and 25°C. (**a**) Incubations concurrent to experiments showed in Fig. 5; (**b**) additional respiration experiment (see text for further details). Lines correspond to linear regression fits.

Contrarily to the pattern observed for feeding and egg production, in the concurrent respiration experiment, the Arrhenius plots for respiratory carbon losses showed that the prolonged thermal exposure caused an increase in the slope (i.e. stronger response; Fig. 6a). This resulted in a shift in Q_10_ coefficient from 1.2 in the short-term response to 1.4 in the medium-term one. The two-tailed test contrasting slopes, however, did not prove significant (F test, *P* = 0.131). The additional respiration experiment carried out with a different batch of females also revealed an increase in slope (i.e. higher Q_10_) after the longer exposure (Fig. 6b; F test, one-tailed Bonferroni adjusted *P* = 0.028). The regression analysis of the pooled respiration data sets was conducted after centring each data set by subtracting the respective arithmetic means. The pooled analysis provided more robust results, further confirming a significant change in slope after the prolonged exposure (F test, *P* = 0.008). The calculated Q_10_ coefficients for the pooled respiration data sets were 1.3 and 1.6 for the short- and medium-term treatments, respectively.

The differences among Q_10_ coefficients for different rate processes depended on time exposure to thermal stress. Thus, after the short-term exposure, the Q_10_ for respiration (pooled value) was significantly lower than those for feeding and egg production (F tests, Bonferroni adjusted *P* < 0.003 in both cases), whereas the Q_10_ values for the latter two did not differ between them (F test, Bonferroni adjusted *P* = 1). Contrarily, no significant differences were revealed among the Q_10_ coefficients for feeding, egg production and respiration after the medium-term exposure (F tests, Bonferroni adjusted *P* = 1 in all cases).

### Stoichiometric ratios, enzymatic biomarkers and LPO levels

**Fig. 7:**
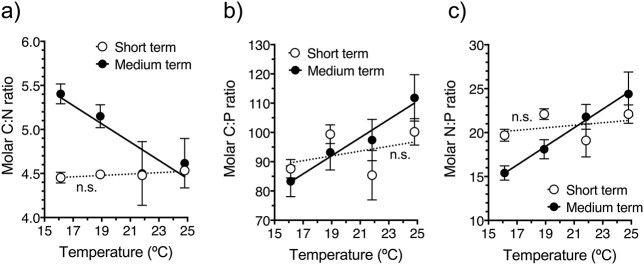
Body stoichiometric ratios of *P. grani* after short- and medium-term exposures at 16, 19, 22 and 25°C. (**a**) C:N, (**b**) C:P, and (**c**) N:P. Notice that these samples were taken at the start of the physiological incubations, and hence the actual exposure was 1 day shorter than for rate processes. Error bars are SE. Lines correspond to linear regression fits. n.s.: not significant.

Fig. 7 shows the molar C:N, C:P and N:P ratios of *P. grani* after short and medium-term thermal exposure. Short-term stress did not result in significant changes in the stoichiometric ratios (linear regression, *P* > 0.19; Fig. 7). Contrarily, the prolonged exposure resulted in a decrease for C:N (linear regression, *P* = 0.019) and an increase for C:P and N:P ratios (linear regression, *P* = 0.020 and 0.002, respectively; Fig. 7).

**Fig. 8:**
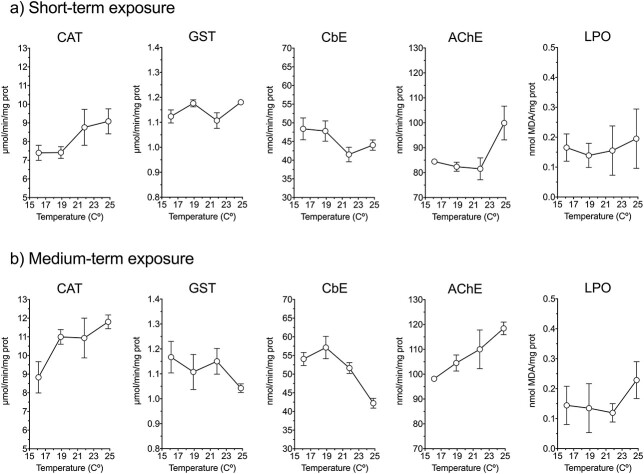
Enzymatic biomarkers and LPO levels of *P. grani* after short- and medium-term exposures at 16, 19, 22 and 25°C. Notice that these samples were taken at the start of the physiological incubations, and hence the actual exposure was 1 day shorter than for rate processes. Error bars are SE.

Among the biomarkers analyzed, only CAT showed a statistically significant increasing trend with temperature after short-term exposure (Fig. 8; linear regression on ln transformed data; *P* = 0.031). The CbE activity showed a negative, but not significant trend (ln transformed; *P* = 0.107), whereas AChE only showed a sharp increase in activity at 25°C. The GST activity and the LPO levels showed no clear trend. Nonetheless, more evident patterns manifested after prolonged exposure. Thus, the CAT and AChE activities were positively related with temperature after medium-term exposure (ln transformed; respectively, *P* = 0.023 and *P* = 0.004), whereas CbE showed the opposite trend (ln transformed; *P* = 0.004). The GST activity and the LPO levels showed no significant relation with temperature (ln transformed; *P* > 0.19 in both cases). The LPO levels at 25°C appeared relatively high (Fig. 8), but they did not significantly differ from the 19°C treatment (t-test, *P* > 0.41).

## DISCUSSION

### Thermal performance curves

We have determined the thermal tolerance and performance of *P. grani* females over a broad range of temperatures and at different temporal scales. At short exposure times (1–2 days, acute responses), *P. grani* showed large phenotypic plasticity and elevated resistance to high temperatures. Survival was challenged at temperatures around 33°C and above, particularly during the second 24-h period and under starvation. Recent studies have reported latitudinal clines in the tolerance to acute (24 h) thermal stress in populations of the copepod *Acartia tonsa* (Sasaki *et al.*, 2019; Sasaki and Dam, 2019). The observed median lethal temperatures of *P. grani* were very similar to those reported for the low-latitude (warm waters) populations of *A. tonsa* by Sasaki and Dam (2019). This fact could be related to the relatively warm rearing conditions (19°C) of our parental *P. grani*. Moreover, the capability of our culture to withstand a 10°C thermal shock, at least for the set time exposure, was noticeable, as it has been reared for several years in a stable thermal environment. This behavior reflects the inherent ability to cope with the short-term variability that characterizes the shallow water bodies and coastal environments where *P. grani* inhabits.

We did not find any relevant effect on *P. grani* survival at temperatures < 33°C; only at the lowest temperature some reduced motility was perceived. At those temperatures, much longer exposure periods would probably be needed to observe any detrimental effect on survival. Median survival times between 16 and 65 days have been reported for feeding females of *P. grani* (Saiz *et al.*, 2015); under starvation, this value diminishes to 6 days at 19°C (E. Saiz, unpub. data). Therefore, it is reasonable to think that even if not feeding, the body reserves could have supported long survival times at the lowest temperatures tested. Contrarily, at very high temperatures, when copepod’s survival is compromised, the boosted metabolic expenditure by temperature may decrease the individual’s tolerance if food is not available. This fact is in agreement with previous evidence that the effects of food limitation in copepod lifespan were temperature dependent (Saiz *et al.*, 2015). Our findings indicate the need to take into account exposure time and food presence for a better assessment of copepod thermal tolerance, particularly at high temperatures.

In contrast to survival curves, physiological performance can be more informative of the susceptibility of a species’ population to thermal stress and its fitness capability within the thermal tolerance range (Pörtner, 2002; Sokolova *et al.*, 2012). In our experiments, this discrepancy was manifested in the acute responses at temperatures beyond 27°C, where sublethal detrimental effects in feeding and fecundity quickly appeared, although the individuals’ survival was not yet compromised. Our data may also suggest a lower thermal optimum for feeding (peaks appeared a bit shifted in Fig. 1c and d). Nevertheless, pellet rates are a rough proxy for feeding, and variations in food assimilation and pellet size and compactness may be responsible for this.

### Thermal exposure, acclimation response and Q_10_ coefficients

The acute thermal stress effects exhibited in ectotherms are considered a passive response following the Arrhenius law, whereas prolonged exposures can result in phenotypic changes to compensate for temperature effects on metabolism (i.e. acclimation response; Schulte *et al.*, 2011; Havird *et al.*, 2020). These longer exposure-driven changes could result in Q_10_ coefficients utterly different from those derived from acute effects (Havird *et al.*, 2020). Accordingly, we found that the magnitude of the temperature-driven effects on *P. grani* feeding and egg production rates declined after prolonged exposures (Fig. 3). Consequently, the Q_10_ coefficients for the above-mentioned rate processes decreased from 2.6–2.7 after acute exposure (2 days) to 1.6–1.7 after the medium-term exposure (7 days). This leveling off pattern was also consistent with that observed in the thermal performance curve experiments (Fig. 2).

The effects of the acclimation response on the Q_10_ coefficients for respiration, however, were somewhat unexpected and acted in the opposite direction (higher Q_10_ after prolonged exposure). We think that the low Q_10_ for respiration under short-term thermal exposure (pooled valued, Q_10_ = 1.3) was a consequence of the interaction of the acute thermal stress (up to 25°C) and exposure time (2 days) with the starving conditions during the incubation. It is acknowledged that the use of filtered seawater significantly diminishes copepod respiration rates (Almeda *et al.*, 2011) and may also quickly shutdown other physiological rates (e.g. fecundity, Saiz *et al.*, 1998). The prolonged thermal exposure (7 days) allowed for metabolic re-equilibration and the Q_10_ values for respiration increased and, even if taking place in filtered seawater, reached values closer (pooled value, Q_10_ = 1.6) to those of the acclimated feeding and egg production Q_10_ coefficients.

It is also worth mentioning at this point that when analyzing the data, we spotted some unexpected variability in the behavior of the parental 19°C treatment. This was particularly evident for the copepod feeding rates (compare 19°C treatments in Fig. 3a). The exact nature of this variability is inexplicit and could be a consequence of uncontrolled differences in the conditions during the experimental run or to intrinsic variability within the copepod population or even at the individual level. In fact, variations on the vital rates of copepods along their lifespan, even at short-time scales, have been reported (e.g. Ceballos and Kiørboe, 2011; Saiz *et al.*, 2015). The use of regression analysis to assess thermal dependence and the acclimation response (change in Q_10_) allowed to elude this issue, since within each trial those unknown sources of variation would be the same.

Regarding the initial hypothesis of temperature-driven mismatch in metabolic rate processes (Alcaraz *et al.*, 2014; Grote *et al.*, 2015), the similarity of the fecal and egg production thermal performance curves of *P. grani* (Fig. 1c and d) does not support it. Furthermore, the Q_10_ coefficients for feeding and reproductive activity did not differ after the short-term exposure; after the prolonged exposure, they both decreased but still were similar and even did not differ statistically from the acclimated Q_10_ coefficient for respiration. The overall lack of mismatch in the thermal responses of *P. grani* is also reflected in the carbon gross-growth efficiencies, which did not show any significant trend with temperature. Had there been a mismatch, we would have expected gross-growth efficiencies to diminish when homeostasis could be compromised. We should have probably extended our combined metabolic experiments to more challenging thermal stress (e.g. 28–30°C) to observe more drastic effects.

The high phenotypic plasticity and notable acclimation capacity of *P. grani* agree with its geographical and seasonal distribution. This species inhabits eastern Atlantic waters, covering a broad latitudinal cline, and also the Mediterranean Sea. It is typically more abundant when waters are warmer, although those temperatures may show geographical variation (Villate, 1982). In this regard, it is worth mentioning that our study has addressed only short- and medium-term direct effects of thermal stress, in the time frame of up to 7 days, and with a thermal amplitude (up to 6°C) that copepods may rarely experience in nature in such period of time. Nevertheless, historical and present records of marine heatwaves show that event durations in the order of 10 days and thermal amplitudes of 2–3°C are common (Hobday *et al.*, 2016; Oliver *et al.*, 2018). Thus, we believe that the processes underlying the phenotypic plasticity observed in our experiments, with all the caution when extrapolating from the laboratory to nature, could probably mimic the conditions faced in such warming episodes. When confronting a gradual rise in ocean temperature at much longer time scales, the effects on copepod species distribution will be modulated by multigenerational and adaptive processes, together with the interaction with biotic and abiotic environmental factors that may also be affected by climate change (e.g. Thor and Dupont, 2015; Isari *et al.*, 2016).

The reduction in thermal effects observed after acclimation has relevant implications for the forecasting of copepod rate-process responses to warming and climate change. Mechanistic models of copepod population dynamics often use high values of the Q_10_ coefficients for both feeding (e.g. 2.5, Banas *et al.*, 2016; 2.6, Dzierzbicka-Glowacka *et al.*, 2010; 2.8, Brun *et al.*, 2019) and respiration (e.g. 2.5, Banas *et al.*, 2016; 3.16, Buitenhuis *et al.*, 2006; 3.4, Carlotti and Wolf, 1998), possibly reflecting that the attenuation effect of acclimation might not have been contemplated (but see Stegert *et al.*, 2009; Acheampong *et al.*, 2014). We have investigated this issue by reviewing published experimental works addressing the effects of temperature on copepod vital rates (Supplementary Table S2). Q_10_ coefficients for the feeding, egg production and respiration rates reported in Supplementary Table S2 averaged, respectively, 2.8 (1.9–3.8 95% confidence interval [CI]), 4.2 (3.5–5.0 95% CI) and 2.8 (2.4–3.2 95% CI). Most of these experimental studies generally lacked a previous exposure period, or it was short (up to 24 h, Supplementary Table S2). This procedure likely prevented the development of the acclimation response, and the rates obtained mostly expressed an acute response, which may undoubtedly overestimate the temperature effects at longer time scales. Our empirical evidence in *P. grani* manifests an urgent need for a better comprehension of copepod phenotypic plasticity and the acclimation response when predicting temperature effects on rate processes, and also a careful re-evaluation of commonly-used Q_10_ coefficients in modeling copepod dynamics.

### Body stoichiometry and biomarkers of oxidative stress

The body stoichiometry of *P. grani* was overall similar to previous reports for the same species (Saiz *et al.*, 2015; Saiz *et al.*, 2020). In our experiments, thermal stress resulted in changes in body stoichiometry only after the medium-term exposure, which presumably reflected new homeostatic values after the acclimation. The decrease in the body C:N ratios of *P. grani* at high temperatures was probably associated with the consumption of body lipid reserves and is in agreement with the observed increase in respiratory carbon losses. Contrarily, body C:P and N:P ratios increased with temperature, indicating a lower relative content of P. Variations in the P content of copepods are typically associated with changes in their RNA content, a P-rich compound and in their growth/activity (Malzahn and Boersma, 2012). However, this is not necessarily the case when different temperatures are involved. Thus, Saiz *et al.* (1998) reported that, because of the Arrhenius effect, the increase in egg production rates of *P. grani* at high temperatures was not accompanied by larger amounts of RNA; similar results were obtained for *Calanus finmarchicus* by Wagner *et al.* (2001). Therefore, it is not surprising that C:P and N:P ratios of *P. grani* increased at high temperatures, since less RNA (and therefore relatively less P) might be required to achieve a given egg production. To our knowledge, very few studies have addressed the changes in copepod body stoichiometry because of warming. Contrarily to our observations, Mathews *et al.* (2018) reported that high temperatures were associated with a decline in the body C:P ratio of the juvenile stages of the copepod *Parvocalanus crassirostris*, although this pattern was not always linked to changes in somatic growth and might be affected by P availability. Hence, temperature may alter the balance in the individual’s requirements of C, N and P, and ultimately will determine, together with resource availability and life-history constraints, the body stoichiometry (Malzahn *et al.*, 2016; Mathews *et al.*, 2018; Saiz *et al.*, 2020).

As noted in the previous section, the physiological rates of the parental 19°C copepod population were not as stable as expected. This issue was also evident in the copepod body stoichiometry, particularly in the C:N ratios. In this case, the copepod C:N ratios at 19°C were quite different between the short- and medium-term exposures, and probably reflected the previous life history of the specimens. The short-term C:N ratios of the copepods were determined at the beginning of the physiological incubations, i.e. after 1-day exposure, and might still be influenced by the food conditions in the rearing tanks, which were high but probably more variable. After longer exposure (6 days) with excess of food, in more strictly controlled conditions, the C:N ratio comparatively increased, possibly reflecting an accumulation of lipids (often found in *Acartia*-like copepods as tiny droplets when food is plentiful). Age-related changes may also help explain variations in copepod body stoichiometry (Saiz *et al.*, 2015). Nevertheless, the statistical comparisons based on the slopes of the thermal relationships, instead of pairwise comparisons for a given temperature across time, allowed to avoid this nuisance.

The use of enzymatic biomarkers of oxidative stress in copepods has recently been developed concerning environmental stress (Vehmaa *et al.*, 2013; Glippa *et al.*, 2018) and copepod aging biology (Saiz *et al.*, 2015). At the cell level, there is a delicate balance between the production of reactive oxygen species and the antioxidant defenses or other cell-repair mechanisms to maintain the redox homeostasis and prevent cell damage (Monaghan *et al.*, 2009). One of the most frequent oxidative damage biomarkers is the measure of LPO levels. They result from the interaction of reactive oxygen species with polyunsaturated fatty acids, compromising the functionality and integrity of the cell membrane (Monaghan *et al.*, 2009). The higher metabolism (and hence higher oxygen consumption rate) boosted by high temperatures is expected to enhance the production of reactive oxygen species at the cell level (Bagnyukova *et al.*, 2007) and, if not mitigated, cause increased levels of LPO. Thus, Vehmaa *et al.* (2013) have reported a 23% increase in LPO levels of the copepod *Acartia bifilosa* associated with a 3°C rise in temperature.

In our experiments we were not able to prove an increase in LPO levels associated with temperature, as we might have expected if the raise in temperature had intensified the production of reactive oxygen species at the cell level and, consequently, oxidative stress. We cannot discard, however, that the high variance among replicates in the LPO analysis, perhaps due to sensitivity constraints, may have precluded the discerning of a positive trend. Moreover, the lack of undeniable increase in LPO levels could also be the consequence of the effectiveness of *P. grani* antioxidant defenses and cell-repair mechanisms at the non-lethal temperatures of our observations (16–25°C), counteracting the action of reactive oxygen species and avoiding cell damage as LPO occurrence (Monaghan *et al.*, 2009). We observed significant changes associated with temperature in the activity of some of the studied enzymatic biomarkers, particularly after the medium-term exposure. Thus, CAT, one of the major antioxidant enzymatic defenses in animal cells (Monaghan *et al.*, 2009), increased with temperature, and this trend strengthened after the prolonged exposure. Contrarily, GST did not clearly respond to the acute thermal stress, and at longer exposures declined with increasing temperature. It is known that biomarker responses are time dependent and may decline over time as a result of either exhaustion or acclimation to exposure conditions (Madeira *et al.*, 2016). Moreover, as mentioned before, biomarkers were determined at temperatures far from the lethal limits, and hence we might not necessarily trigger strong responses in our experimental organisms. Similarly, CbE responses of *P. grani* in our experiments were diminished under medium-term exposure at elevated temperatures. The CbE biomarker is commonly used in ecotoxicology given its detoxification capacity (Nos *et al.*, 2021), and our results suggest that the copepod’s capability to face environmental chemical stressors (CbE, and also GST has this function) may be compromised at high temperatures, therefore challenging its health status under a forecasted contaminant threat (Noyes *et al.*, 2009). Finally, the variation of AChE was in line with temperature increases, as found in other animal models (Pfeifer *et al.*, 2005; Pallarés *et al.*, 2020), and could be regarded as a natural physiological response, within the range of thermal tolerance, to changes in feeding and locomotor activity (Xuereb *et al.*, 2009). Overall, the variation in the biomarker activities agrees with the acclimation response (phenotypic plasticity) we have observed in *P. grani* at the individual level (vital rates, i.e. feeding, egg production, respiration). The molecular and enzymatic defense systems of *P. grani* appear suited to allow this species to cope well with the thermal stress we forced in these experiments (6°C increase concerning the control 19°C temperature), with no significant mortality and still reproducing at high rates (Note: in our lab, this copepod species has been kept for >20 generations at 25°C, which further confirms this).

## CONCLUSIONS

Our study has shown that the copepod *P. grani* has high phenotypic plasticity and substantial acclimation capacity to temperature, which helps explain its broad distribution. In addition to the study of survival curves, assessing the effects of temperature on the performance of key physiological processes, like feeding, reproduction, or maintenance costs, can provide a better proxy for the species capability to cope with a moderate (i.e. within tolerance limits) temperature change. Contrarily to expected, we did not find a mismatch between the thermal responses of the studied physiological rates, being the Q_10_ values for feeding and reproduction similar. The acclimation response in the organismal rate processes was exposure dependent, and resulted in the flattening of physiological rates and a shift to lower Q_10_ coefficients. This response was accompanied by changes at the cell level on the antioxidant defense and cell-repair mechanisms to avoid oxidative cell damage. The interest of the thermal acclimation response in copepods goes beyond the mere physiological relevance. Many numerical models of copepod population dynamics use high Q_10_ coefficients to predict copepod activity under different temperature scenarios. However, the examination of published studies on direct effects of temperature on copepod vital rates evidences the lack or disparity on acclimation periods, raising cautious concerns on the use of such high Q_10_ values for predicting non-acute effects on copepod rate processes.

## Supplementary Material

REDUCTION_IN_THERMAL_STRESS_JPR_Supplementary_fbac017

## Data Availability

The data will be deposited in the DIGITAL.CSIC repository after publication.
