# Supplementary material for: Reduction in thermal stress of marine copepods after physiological acclimation
Source: J Plankton Res. 2022 Apr 8;44(3):427–42. doi: 10.1093/plankt/fbac017 (PMC9155217; doi:10.1093/plankt/fbac017)
Supplement: REDUCTION_IN_THERMAL_STRESS_JPR_Supplementary_fbac017 [file reduction_in_thermal_stress_jpr_supplementary_fbac017.pdf]

SUPPLEMENTARY FIGURES

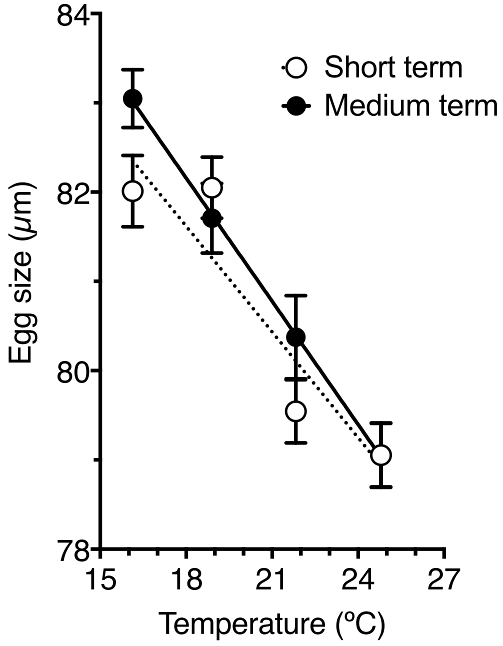

Supplementary Fig. S1. *P. grani* egg diameter as a function of temperature and exposure duration (short term: 2 d; medium term: 7 d). Parental population temperature was 19ºC. Error bars are SE.

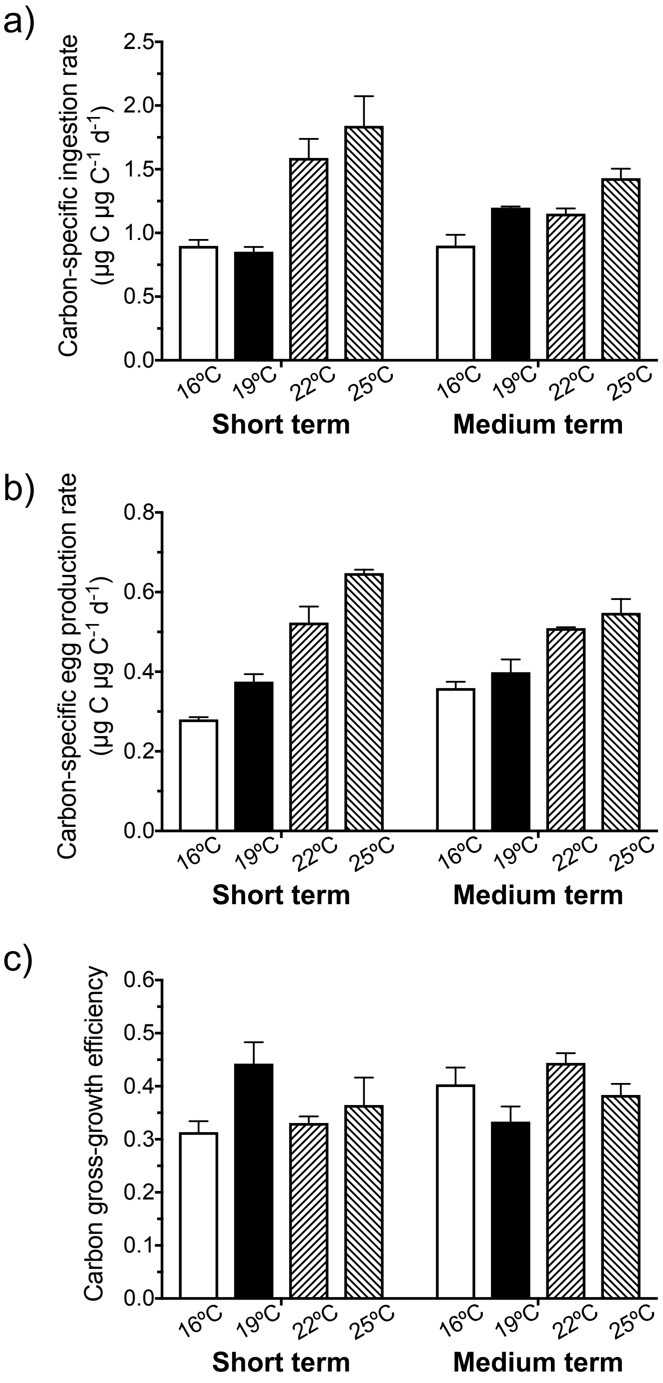

Supplementary Fig. S2. Carbon-specific feeding (a) and egg production (b) rates, and carbon gross-growth efficiency (c) of *P. grani* after short-term and medium-term exposures at 16, 19, 22, and 25ºC. Error bars are SE.

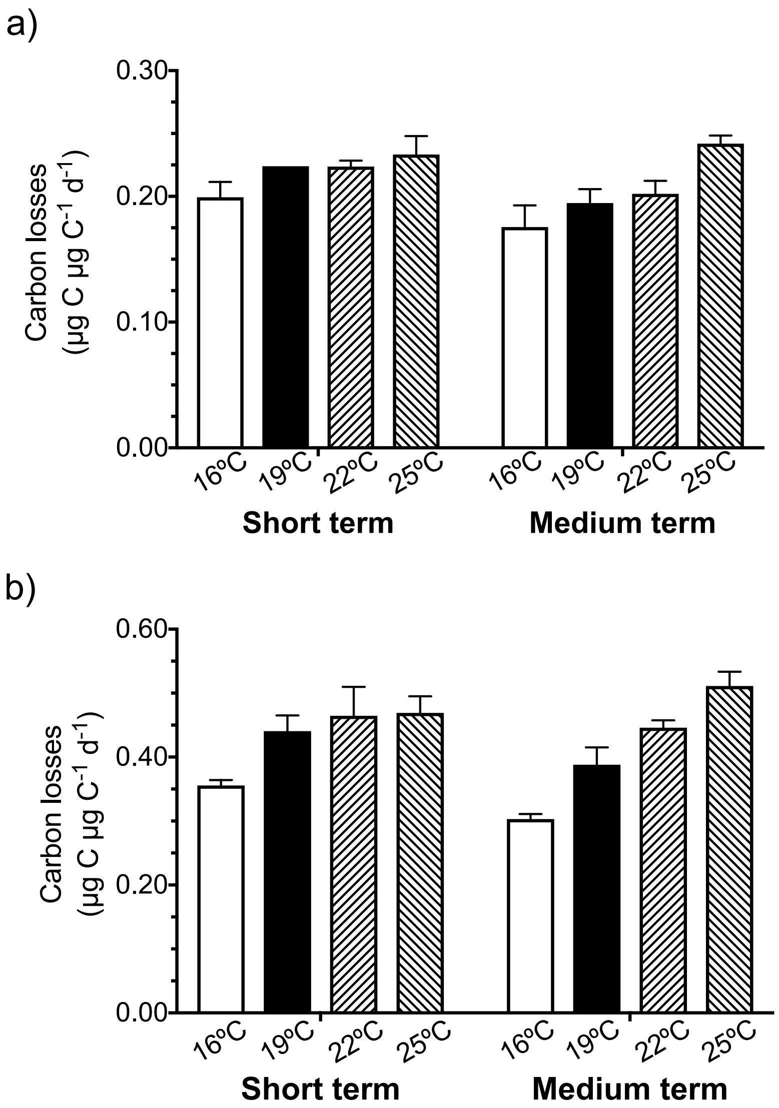

Supplementary Fig. S3. Respiratory carbon losses of *P. grani* after short- and medium-term exposures at 16, 19, 22, and 25ºC. a) first trial, concurrent with feeding and egg production experiments; b) additional experiment with a different copepod cohort. Error bars are SE.

SUPPLEMENTARY TABLES

Supplementary Table S1. Activation energy values (E_a, eV) for the different variables and time exposures obtained from the slopes of the Arrhenius plots, converted into eV units using the Boltzmann constant (8.6173×10^{-5} eV K^{-1}).

	**Short term (2 d)**	**Medium term (7 d)**
**Carbon-specific ingestion rate (μg C μg C^{-1} d^{-1})**	0.70	0.35
**Carbon-specific egg production rate (μg C μg C^{-1} d^{-1})**	0.73	0.39
**Respiratory carbon losses (μg C μg C^{-1} d^{-1})**	0.13	0.26
**Respiratory carbon losses (μg C μg C^{-1} d^{-1}) – Second trial**	0.22	0.42
**Respiratory carbon losses (μg C μg C^{-1} d^{-1}) – Pooled**	0.17	0.35

Supplementary Table S2. List of Q_{10} coefficients for egg production, feeding and respiration rates of marine copepods. Acclimation conditions are also provided. Q_{10} coefficients have been recalculated from original papers fitting a simple linear regression line to ln-transformed rate data as a function of temperature, and then $Q_{10} = \exp(10 \times \text{slope})$. When within a study there was more than one dataset for a given species, we provide mean Q_{10} value among datasets, range of values and sample size. Occasionally, flattening or decline of physiological rates were reported after certain threshold temperature, and Q_{10} was computed for the ascending part of the dataset only.

**COPEPOD SPECIES**	**RATE PROCESS**	**ACCLIMATION**	**EXPONENTIAL Q_{10}**	**SOURCE**
Acartia clausi	egg production	None or 2-3ºC/day	3.43 (2.45–4.40, 2)	Uye 1981
Acartia hudsonica	egg production	No[1]	2.52	Durbin et al 1992
Acartia steueri	egg production	None or 2-3ºC/day	6.23	Uye 1981
Acartia tonsa	egg production	Weeks[2]	4.05	Corkett & Zillioux 1975
Calanus finmarchicus	egg production	10-21 days[3]	5.43	Hirche et al 1997
Centropages hamatus (North Sea)	egg production	24 hours[4]	5.14	Halsband-Lenk et al 2002
Centropages typicus	egg production	1 day	4.38	Saiz et al 1999
Centropages typicus (Mediterranean)	egg production	24 hours[4]	3.54	Halsband-Lenk et al 2002
Centropages typicus (North Sea)	egg production	24 hours[4]	4.30	Halsband-Lenk et al 2002
Paracartia grani	egg production	2 days	4.63	Saiz et al. 1998
Pseudocalanus elongatus	egg production	Weeks[2]	2.85	Corkett & Zillioux 1975
Temora longicornis	egg production	Weeks[2]	3.18	Corkett & Zillioux 1975
Temora longicornis (North Sea)	egg production	24 hours[4]	2.95	Halsband-Lenk et al 2002
Temora stylifera (Mediterranean)	egg production	24 hours[4]	6.76	Halsband-Lenk et al 2002
Acartia hudsonica	ingestion	No[5]	2.12	Durbin & Durbin 1992
Acartia tonsa	ingestion	3 days	6.57 (6.20–6.94, 2)	Tyrell & Fischer 2019
Calanus helgolandicus	ingestion	4-6 hours	1.92 (1.60–2.13, 3)	Fernández 1978
Centropages hamatus	ingestion	<3 days[6]	3.27	Kiørboe et al 1982
Centropages typicus	ingestion	No[6]	5.06	Sell et al. 2001

Clausocalanus acuicornis	ingestion	4-6 hours	1.92	Fernández 1978
Neocalanus robustior	ingestion	4-6 hours	1.66	Fernández 1978
Oithona davisae	ingestion	3 hours	2.39	Almeda et al 2010
Parvocalanus crassirostris	ingestion	3 days	1.81 (1–2.61, 2)	Tyrell & Fischer 2019
Pleuromamma gracilis	ingestion	4-6 hours	2.34	Fernández 1978
Temora stylifera	ingestion	4-6 hours	2.17 (1.58–3.19, 6)	Fernández 1978
Temora stylifera	ingestion	No	2.46	Thébault 1985
Acartia clausi	respiration	No	1.83	Champalbert & Gaudy 1972
Acartia clausi	respiration	4-6 hours	2.34 (2.21–2.47, 2)	Fernández 1978
Acartia clausi	respiration	No	2.06 (1.43–3.57, 11)	Gaudy 1973
Acartia clausi	respiration	No[7]	1.95	Gaudy et al 2000
Acartia clausi	respiration	No	1.78	Gauld & Raymont 1953
Acartia clausi	respiration	No	1.77 (1.71–1.82, 2)	Nival et al 1974
Acartia tonsa	respiration	No[8]	1.5	Gaudy et al 2000
Anomalocera patersoni	respiration	No	2.70 (2.14–3.49, 3)	Champalbert & Gaudy 1972
Anomalocera patersoni	respiration	4-6 hours	1.95 (1.90–1.99, 2)	Fernández 1978
Calanoides acutus	respiration	No	3.96	Hirche 1984
Calanus finmarchicus	respiration	No	2.76	Clarke & Bonnet 1939
Calanus finmarchicus	respiration	No	3.24	Hirche 1987
Calanus finmarchicus	respiration	No	2.08 (1.74–2.26, 3)	Marshall et al 1935
Calanus glacialis	respiration	No	4.49	Hirche 1987
Calanus helgolandicus	respiration	No	2.05	Champalbert & Gaudy 1972
Calanus helgolandicus	respiration	4-6 hours	3.67 (2.36–6.28, 5)	Fernández 1978
Calanus helgolandicus	respiration	No	1.87 (1.58–2.20, 3)	Gaudy 1973
Calanus helgolandicus	respiration	No	2.84 (1.67–4.00, 2)	Nival et al 1974
Calanus hyperboreus	respiration	No	2.26 (2.15–2.37, 2)	Hirche 1987
Calanus pacificus	respiration	Since CI stage	1.82	Vidal 1980
Candacia ethiopica	respiration	No	2.85	Gaudy 1975

Centropages chierchiae	respiration	24 hours	2.74 (2.72–2.75, 2)	Cruz et al 2013
Centropages hamatus	respiration	No	2.70	Gauld & Raymont 1953
Centropages hamatus	respiration	No	2.35 (1.58–3.12, 2)	Raymont 1959
Centropages typicus	respiration	No	2.77	Champalbert & Gaudy 1972
Centropages typicus	respiration	4-6 hours	1.83 (1.75–1.95, 3)	Fernández 1978
Centropages typicus	respiration	No	2.04 (1.41–3.39, 14)	Gaudy 1973
Centropages typicus	respiration	No	2.94	Nival et al 1974
Clausocalanus acuicornis	respiration	4-6 hours	2.94 (2.87–3.00, 2)	Fernández 1978
Eucalanus crassus	respiration	No	3.13	Nival et al 1974
Eucalanus elongatus	respiration	No	6.67	Gaudy 1975
Euchaeta acuta	respiration	No	1.03	Champalbert & Gaudy 1972
Euchaeta acuta	respiration	No	6.65	Nival et al 1974
Euchaeta antarctica	respiration	No	5.23	Hirche 1984
Euchaeta marina	respiration	No	3.37	Gaudy 1975
Euchirella messinensis	respiration	No	1.66	Gaudy 1975
Eurytemora herdmani	respiration	No	1.00	Raymont 1959
Gaetanus kruppii	respiration	No	2.7	Gaudy 1975
Labidocera wollastoni	respiration	No	2.02	Champalbert & Gaudy 1972
Lucicutia lucida	respiration	No	3.7	Gaudy 1975
Metridia longa	respiration	No	2.06	Hirche 1987
Neocalanus robustior	respiration	4-6 hours	1.99 (1.73–2.36, 3)	Fernández 1978
Oithona davisae	respiration	2 hours	2.52	Almeda et al 2011
Oithona similis	respiration	Overnight	3.06	Castellani et al 2005
Paraeuchaeta gracilis	respiration	No	4.03	Gaudy 1975
Pleuromamma abdominalis	respiration	No	2.56	Gaudy 1975
Pleuromamma gracilis	respiration	4-6 hours	2.21 (2.13–2.29, 2)	Fernández 1978
Pleuromamma xiphias	respiration	No	2.54	Champalbert & Gaudy 1972
Pontellopsis regalis	respiration	No	1.85	Champalbert & Gaudy 1972
Pontellopsis villosa	respiration	No	1.41 (1.31–1.51, 2)	Champalbert & Gaudy 1972

Pseudodiaptomus annandalei	respiration	1-2ºC/d + 2-4 days	2.77	Lehette et al 2016
Pseudodiaptomus hessei	respiration	24 hours	2.99 (2.92–3.06, 2)	Isla & Perissinotto 2004
Rhincalanus nasutus	respiration	No	11.52	Champalbert & Gaudy 1972
Temora longicornis	respiration	No	2.47	Gauld & Raymont 1953
Temora stylifera	respiration	No	3.71	Champalbert & Gaudy 1972
Temora stylifera	respiration	4-6 hours	2.54 (1.88–3.42, 4)	Fernández 1978
Temora stylifera	respiration	No	2.14 (1.60–4.08, 9)	Gaudy 1973
Temora stylifera	respiration	No	3.08 (1.73–5.64, 4)	Nival et al 1974
Tortanus discaudatus	respiration	No	1.39	Raymont 1959
Undeuchaeta plumosa	respiration	No	2.46	Champalbert & Gaudy 1972

[1]Field animals collected at temperatures similar to the experimental ones
[2]EPR over female lifetime (weeks)
[3]4-7 d acclimation to 0ºC to standardize. Then incubations between 10 and 21 days for each temperature
[4]EPR over a 5 day period
[5]Field animals collected at field temperatures similar (<3ºC) to the experimental ones. 72 h conditioning to food
[6]Not homogenous source: field animals from different samplings
[7]At the habitat salinity (35 ppt)
[8]At the habitat salinity (15 ppt)
